# Nitric oxide‐soluble guanylyl cyclase pathway as a contributor to age‐related memory impairment in *Drosophila*

**DOI:** 10.1111/acel.13691

**Published:** 2022-08-13

**Authors:** Ayako Tonoki, Saki Nagai, Zhihua Yu, Tong Yue, Sizhe Lyu, Xue Hou, Kotomi Onuki, Kaho Yabana, Hiroki Takahashi, Motoyuki Itoh

**Affiliations:** ^1^ Department of Biochemistry, Graduate School of Pharmaceutical Sciences Chiba University Chiba Japan; ^2^ Medical Mycology Research Center Chiba University Chiba Japan

**Keywords:** aging, *Drosophila*, glia, memory, nitric oxide, soluble guanylyl cyclase

## Abstract

Age‐related changes in the transcriptome lead to memory impairment. Several genes have been identified to cause age‐dependent memory impairment (AMI) by changes in their expression, but genetic screens to identify genes critical for AMI have not been performed. The fruit fly is a useful model for studying AMI due to its short lifespan and the availability of consistent techniques and environments to assess its memory ability. We generated a list of candidate genes that act as AMI regulators by performing a comprehensive analysis of RNAsequencing data from young and aged fly heads and genome‐wide RNAi screening data to identify memory‐regulating genes. A candidate screen using temporal and panneuronal RNAi expression was performed to identify genes critical for AMI. We identified the *guanylyl cyclase β‐subunit at 100B* (*gycβ*) gene, which encodes a subunit of soluble guanylyl cyclase (sGC), the only intracellular nitric oxide (NO) receptor in fruit flies, as a negative regulator of AMI. RNAi knockdown of *gycβ* in neurons and NO synthase (NOS) in glia or neurons enhanced the performance of intermediate‐term memory (ITM) without apparent effects on memory acquisition. We also showed that pharmacological inhibition of sGC and NOS enhanced ITM in aged individuals, suggesting the possibility that age‐related enhancement of the NO‐sGC pathway causes memory impairment.

## Graphical abstract

Intermediate‐term memory (ITM) is negatively regulated by nitric oxide (NO) synthesis in glia and expression of soluble guanylyl cyclase (sGC) in neurons. Age‐dependent impairment of ITM can be reversed by inhibition of NOS and sGC, suggesting that overproduction of NO in glia and activation of sGC signaling in neurons causes age‐related memory impairment.

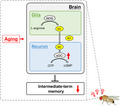

**Abbreviations**
AMI: age‐dependent memory impairment
ARM: anesthesia‐resistant memory
ASM: anesthesia‐sensitive memory
CREB: cAMP response element‐binding protein
GFP: green fluorescent protein
GS: gene‐switch
gycβ: guanylyl cyclase β‐subunit at 100B
ITM: intermediate‐term memory
L‐NAME: N‐nitro‐L‐arginine methyl ester
NO: nitric oxide
NOS: NO synthase
ODQ: 1*H*‐[1,2,4]oxadiazolo[4,3‐*a*]quinoxalin‐1‐one
PKA: protein kinase A
sGC: soluble guanylyl cyclase

## 1 INTRODUCTION

Aging decreases learning and memory formation. A large body of evidence indicates that age‐dependent changes in gene expression lead to brain dysfunction and memory impairment (Bishop et al., 2010; Smith et al., 2020). Transcriptome profiling studies of postmortem human brains (Erraji‐Benchekroun et al., 2005; Lu et al., 2004) and aging brains in various model animals, including flies (Davie et al., 2018; Pacifico et al., 2018), zebrafish (Arslan‐Ergul & Adams, 2014), and mice (Lee et al., 2000; Prolla, 2002), have revealed evolutionarily conserved pathways in age‐related changes in gene expression, such as reduced expression of mitochondrial and neuronal function genes and increased expression of stress response and immune/inflammatory response genes (Aramillo Irizar et al., 2018; Loerch et al., 2008). However, it has been difficult to identify genes that cause AMI in humans or mammalian model organisms since such studies require many aged animals showing memory impairment and consistent techniques and environments to assess their memory ability.

*Drosophila melanogaster* is an ideal system to perform a genetic screen for candidate genes that regulate AMI, particularly because it (i) exhibits robust memory formation with olfactory classical conditioning (Quinn et al., 1974; Tempel et al., 1983; Tully & Quinn, 1985); (ii) shows age‐dependent disturbances in memory (Tamura et al., 2003; Tonoki & Davis, 2012, 2015); (iii) provides powerful genetic tools, including large collections of mutants, RNAi lines, and *Gal4* driver lines; and (iv) has a relatively short lifespan of approximately 2 months. Previous genetic mutant screens of the regulation of olfactory memory in young flies have identified numerous factors, including cAMP phosphodiesterase, calcium/calmodulinactivated adenylyl cyclase, and catalytic subunit of PKA (Byers et al., 1981; Dubnau et al., 2003; Dudai et al., 1976; Quinn et al., 1979). A recent genome‐wide RNAi screen has identified genes critical for memory formation using panneuronal RNAi expression in *Drosophila* (Walkinshaw et al., 2015). In this study, we identified nitric oxide‐soluble guanylyl cyclase (NO‐sGC) as a negative regulator of ITM from a comprehensive analysis of RNA‐sequencing data from young and aged fly heads and published data from a genome‐wide RNAi screen. We further found that inhibition of the NO‐sGC pathway enhanced ITM in both young and aged individuals. These data revealed a critical role for the NO‐sGC pathway in AMI.

## 2 RESULTS

### 2.1 Candidate genes involved in age‐related memory impairment

**FIGURE 1:**
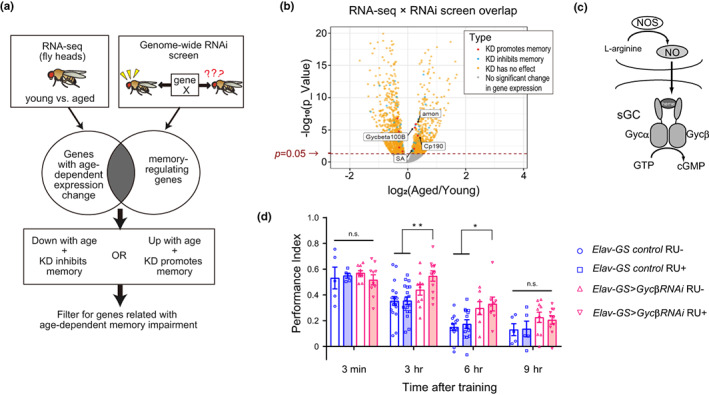
Neuron‐specific knockdown of *gycβ* enhances intermediate‐term memory. (a) The screening strategy to identify genes that regulate memory formation and are differentially expressed with age. (b) Volcano plot showing differential gene expression profiles in the heads of young and aged flies. The plot shows the negative log_10_ of the false discovery rate value (Y‐axis) against log_2_ of the fold change (X‐axis). Significant hits from a genome‐wide RNAi screen were overlaid. The genes whose knockdown promotes memory, inhibits memory, and has no effect, are represented by the red, blue, and orange dots, respectively. The dashed line represents an adjusted *p* value of 0.05. (c) Diagram of NO and soluble guanylyl cyclase (sGC) in *Drosophila*. (d) Olfactory memory assay using the gene switch system. *Elav‐GS > GFP* was used as the control. Transient and neuron‐specific knockdown of *gycβ* (*Elav‐GS > gycβ* ^*RNAi*^) did not affect memory acquisition or 9‐h memory but significantly enhanced 3‐ or 6‐h memory (two‐way ANOVA followed by post hoc Tukey's multiple comparisons test at each time after training. 3 min, *n* = 5, 5, 10, and 10 for control RU‐, control RU+, RNAi RU‐, and RNAi RU+ data respectively. Two‐way ANOVA *F*_(1,26)_ = 0.0045, *p* = 0.95; 3 h, *n* = 18, 18, 10, and 11 for control RU‐, control RU+, RNAi RU‐, and RNAi RU+ data respectively. Two‐way ANOVA *F*_(1,53)_ = 13.95, *p* = 0.0005, post hoc Tukey's multiple comparisons test, **p < 0.005, control RU‐ RU+ versus RNAi RU+; 6 h, *n* = 13, 13, 7, and 8 for control RU‐, control RU+, RNAi RU‐, and RNAi RU+ data respectively. Two‐way ANOVA *F*_(1,37)_ = 14.27, *p* = 0.0006, post hoc Tukey's multiple comparisons test, *p < 0.05, control RU‐ RU+ versus RNAi RU+; 9 h, *n* = 5, 5, 10, and 10 for control RU‐, control RU+, RNAi RU‐, and RNAi RU+ data respectively. Two‐way ANOVA *F*_(1,26)_ = 3.265, *p* = 0.0824.). n.s., not significant. Data are mean ± SEM

We aimed to identify genes that were differentially expressed between young and aged individuals and that regulated memory formation. To do so, we first identified a set of agedependent upregulated/downregulated genes by performing sequencing of the RNA (RNAseq) from heads in young and aged wild‐type *Canton‐S* flies (Figure 1a). RNA‐seq analysis identified 831 genes as age‐dependent upregulated genes and 1235 genes as agedependent downregulated genes (Figure S1a,b). Second, these gene lists were compared with data from recent genome‐wide RNAi screens to identify genes critical for memory formation using panneuronal RNAi expression in *Drosophila* (Walkinshaw et al., 2015; Figure 1a). The results of the large RNAi screen identified >500 genes as positive memory regulators and 42 genes as negative memory regulators. Finally, we generated a list of genes that overlapped between the two lists of genes from the RNA‐seq data and genomewide RNAi screen data (Figure 1b, Figure S1a,b), which included four genes that were negative‐memory regulators with age‐dependent upregulated gene expression (Figure 1b, Figure S1a, and Table S1) and 54 genes that were positive memory regulators with agedependent downregulated gene expression (Figure S1b and Table S2).

Next, we examined whether these candidate genes regulated memory in neurons without an effect on development or growth in young flies. The candidate genes were transiently knocked down in neurons only after flies were fed RU486 (RU) using the spatiotemporal inducible Gene‐Switch system (*Elav‐GS*; McGuire et al., 2003). Five‐day‐old flies were fed RU‐containing food for 5 days, and then, their ITM performance was tested at 3 h after conditioning. Enhancements in 3‐h memory were observed in flies expressing *cp190* ^*RNAi*^ or *gyc β* ^*RNAi*^ in neurons compared with control flies (Figure S1c).

### 2.2 Genetic knockdown of *gyc* *β* in neurons enhances intermediate‐term memory

*Guanylyl cyclase* *β* *‐subunit at 100B* (*gyc* *β*, *cg1470*) is a subunit of soluble guanylyl cyclase (sGC), the only intracellular NO receptor in fruit flies, and consists of two subunits, Gycα and Gycβ. sGC is activated upon binding to NO, resulting in elevated cGMP levels (Morton et al., 2005) (Figure 1c).

To examine which types of memory are enhanced by knockdown of *gyc β*, we measured memory at various times after conditioning in control flies expressing green fluorescent protein (GFP) and experimental flies transiently expressing *gyc β* ^*RNAi*^ in neurons. Although significant leaky expression was observed without RU feeding, the knockdown efficiency of *gyc β* was approximately 50% with RU feeding (Figure S2a). The ITM formed after singlecycle conditioning is classified into two distinct phases, anesthesia‐sensitive memory (ASM) and anesthesia‐resistant memory (ARM) (Quinn and Dudai, 1976). Usually, 3‐h memory after single conditioning comprises ASM and ARM; however, 6 h after single conditioning, ASM is almost decayed and most of the memory is ARM (Davis, 2005; Heisenberg, 2003). We found that memory performance was significantly enhanced at 3 or 6 h, but not at 3 min or 9 h after conditioning in experimental flies (Figure 1d). The enhanced 3‐ or 6‐h memory in flies expressing *gyc β* ^*RNAi*^ in neurons was not attributable to an enhanced ability to perceive the shock and odors used during the experiments (Figure S2b,c). Furthermore, after cold shock application that eliminates only ASM, significant enhanced 3 h memory was observed in flies expressing *gyc β* ^*RNAi*^ in neurons (Figure S2d). However, the degree of ARM enhancement in the *gyc β* ^*RNAi*^ group was smaller than that of memory enhancement in the absence of cold shock (Figure 1d). Taken together, our data suggest that *gyc β* in neurons has a negative effect on ITM, specifically on ARM but also ASM, but leave open the effect on LRM formation.

### 2.3 Pharmacological inhibition of soluble guanylyl cyclase enhances intermediate‐term memory

**FIGURE 2:**
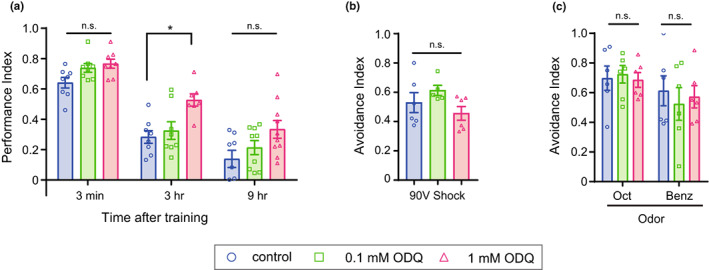
Pharmacological inhibition of sGC enhances intermediate‐term memory. (a) Olfactory memory assay in young flies fed ODQ, an inhibitor of sGC. ODQ administration significantly improved 3‐h memory, while it had a relatively small effect on 3‐min or 9‐h memory (two‐way ANOVA followed by post hoc Tukey's multiple comparisons test; 3 min, *n* = 8 for each; 3 h, *n* = 8, 8, and 7 for control, 0.1 mM ODQ, and 1 mM ODQ; 9 h, *n* = 8, 9, and 10 for control, 0.1 mM ODQ, and 1 mM ODQ. Two‐way ANOVA *F*_(2,65)_ = 12.29, *p* < 0.0001, post hoc Tukey's multiple comparisons test, *p = 0.0201, 3 h control versus 3 h 1 mM ODQ). (b) Shock avoidance test in flies fed ODQ. The experimental group and control group showed no significant difference in shock avoidance (one‐way ANOVA followed by post hoc Tukey's multiple comparisons test. *n* = 6, 5, and 6 for control, 0.1 mM ODQ, and 1 mM ODQ. Oneway ANOVA *F*_(2,14)_ = 2.049, *p* = 0.1658). (c) Odor avoidance test in flies fed ODQ. The experimental group and control group showed no significant difference in the avoidance of odors, Oct and Benz (two‐way ANOVA followed by post hoc Tukey's multiple comparisons test. *n* = 6 for each data. Two‐way ANOVA *F*_(2,30)_ = 0.08142, *p* = 0.9220). n.s., not significant. Data are mean ± SEM for all

Next, we examined whether the inhibition of sGC enhanced ITM. We fed flies with the sGC inhibitor 1*H*‐[1,2,4]oxadiazolo[4,3‐*a*]quinoxalin‐1‐one (ODQ) for 18 h before conditioning and examined their memory performance. A significant enhancement in memory performance at 3 h after conditioning was observed in a group of flies fed 1 mM ODQ dissolved in 5% sucrose but not those that were fed 0.1 mM ODQ dissolved in 5% sucrose or the control group fed 5% sucrose only (Figure 2a). In contrast, no significant difference in performance was observed between the control and experimental groups at 3 min or 9 h after conditioning (Figure 2a). The memory enhancement in flies fed ODQ was not attributable to an enhanced ability to perceive the shock and odors used during the experiments (Figure 2b,c). These data suggest that sGC affects ITM but not STM.

### 2.4 Expression of *gyc* *β* in mushroom body (MB) α′β′ neurons inhibits memory performance

**FIGURE 3:**
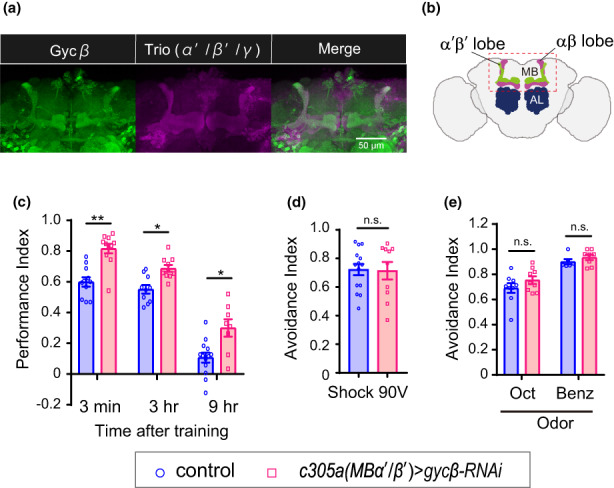
Knockdown of *gycβ* in MB *α′β′* neurons enhances intermediate‐term memory. (a) Fly brains expressing membrane‐tethered GFP (*UAS‐mCD8::GFP*) driven by *Gyc β ‐Gal4* ^*MI01568*^ colabeled with an anti‐TRIO antibody to mark MB α′β′ neurons. (b) an image of the adult brain showing the αβ and α′β′ lobes of the mushroom bodies (MB) and the antennal lobes (AL). (c) MB α′β′ neuron‐specific knockdown of *gycβ* (*c305a > Gyc*β^*RNAi*^) significantly enhanced 3‐min, 3‐, and 9‐h memory compared with control (*c305a > GFP*) (3 min, *n* = 11 for each data, U = 9, **p = 0.0003; 3 h, *n* = 10 for each data, U = 12, *p = 0.0029; 9 h, *n* = 13 and 8 for control and *gycβ* ^*RNAi*^, U = 19, *p = 0.0150, Mann–Whitney U test). (d) Shock avoidance test in *gycβ* knockdown flies. There was no significant difference between the experimental group and the control group in the avoidance of electric shock (n = 14 and 10 for control and *gycβ* ^*RNAi*^, *t*_(22)_ = 0.1168, *p* = 0.9080, unpaired *t* test). (e) Odor avoidance test in *gycβ* knocked‐down flies. There was also no significant difference between the experimental group and the control group in the avoidance of odors, Oct and Benz. (Oct: *n* = 9 for each data, U = 29.5, *p* = 0.3507; Benz: *n* = 6, and 8 for control and *gycβ* ^*RNAi*^, U = 18, *p* = 0.4872). n.s., not significant. Data are mean ± SEM for all

To identify the types of neurons expressing *gyc β* to regulate memory, we examined the brains of flies expressing a membrane‐tethered GFP (*UAS‐mCD8::GFP*) driven by *Gyc β ‐Gal4* (Diao et al., 2015). Projections of confocal stacks revealed strong GFP expression in MB α′β′ neurons, which was shown by colabeling brains with an anti‐TRIO antibody to mark the MB α′β′ neurons (Figure 3a,b). Weak GFP expression in MB αβ neurons was shown by colabeling brains with an anti‐FASII antibody which marks the MB αβ neurons (Figure S3a and Figure 3b). A similar expression pattern was also observed in flies expressing a protein trap.

Gycβ‐EGFP fusion protein (MI08892; Venken et al., 2011) (Figure S3b, control, left panels). To investigate the role of Gycβ specifically in MB α′β′ neurons, we measured memory after conditioning in control flies expressing *gfp* and experimental flies expressing *gyc β* ^*RNAi*^ in MB α′β′ neurons using the MB α′β′ neuron Gal4 driver *c305a‐Gal4* (Figure 3c). Gycβ‐EGFP signals in the MB α′β′ neurons were significantly reduced with the expression of *gyc β* ^*RNAi*^ in the MB α′β′ neurons using *c305a‐Gal4* (Figure S3b, right panels, Figure S3c). Memory performance measured at 3 min, 3, and 9 h after conditioning was enhanced without any effect on the ability to perceive shock and odors (Figure 3c–e). Furthermore, in flies expressing *gyc β* ^*RNAi*^ in MB α′β′ neurons using *MB005B‐Gal4* or *MB463B‐Gal4* (Aso et al., 2014), which is a more specific MB α′β′ neuron Gal4 driver than *c305a‐Gal4*, enhanced memory measured at 3 h was observed without a significant effect on the ability to perceive shock and odors (Figure S4a–g). These data suggest that inhibition of sGC in MB α′β′ neurons nonspecifically enhances ITM, but also could enhance STM when inhibited from developmental stage using the *Gal4/UAS* system.

### 2.5 Pharmacological inhibition of NOS enhances intermediate‐term memory

**FIGURE 4:**
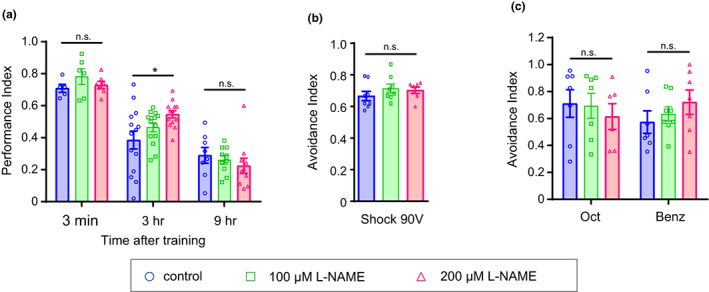
Pharmacological inhibition of NOS enhances intermediate‐term memory. (a) Olfactory memory assay in young flies fed L‐NAME, an inhibitor of NOS. L‐NAME administration significantly enhanced 3‐h memory but did not affect 3‐min or 9‐h memory (3 min, *n* = 5, 6, and 7 for control, 100 μM L‐NAME, and 200 μM L‐NAME, *p* = 0.5623, Kruskal–Wallis test; 3 h, *n* = 14, 14, and 13 for control, 100 μM L‐NAME, and 200 μM LNAME, *p* = 0.0382, Kruskal–Wallis test, post hoc Dunn's multiple comparisons test, *p = 0.0355, control versus 200 μM L‐NAME; 9 h, *n* = 8, 10, and 10 for control, 100 μM L‐NAME, and 200 μM L‐NAME, *p* = 0.2529, Kruskal–Wallis test). (b) Shock avoidance test in flies fed L‐NAME. There was no significant difference between flies fed L‐NAME and control flies in the avoidance of electric shock (*n* = 8, 8, and 7 for control, 100 μM L‐NAME, and 200 μM LNAME, one‐way ANOVA *F*_(2,20)_ = 0.8992, *p* = 0.4227). (c) Odor avoidance test in flies fed LNAME. There was no significant difference between flies fed L‐NAME and control flies in the avoidance of odors, Oct and Benz (two‐way ANOVA *F*_(2,36)_ = 0.05308, *p* = 0.9484. Oct, *n* = 7, 7, and 6; Benz, *n* = 7, 8, and 7 for control, 100 μM L‐NAME, and 200 μM L‐NAME respectively). n.s., not significant. Data are mean ± SEM for all

We next investigated whether the negative effect of sGC on ITM was because of increased NO levels (Figure 1c). NO levels can be modulated pharmacologically by feeding flies with the NOS inhibitor N‐nitro‐L‐arginine methyl ester (L‐NAME) for 18 h. A significant enhancement in memory performance at 3 h, but not at 3 min or 9 h, after conditioning was observed in flies fed 200 μM L‐NAME (Figure 4a). The enhancement in 3‐h memory in flies fed L‐NAME was not attributable to an enhancement in shock and odor sensitivity (Figure 4b,c). These data suggest that NO has a negative effect more on ITM than STM.

### 2.6 NOS expression in glia inhibits intermediate‐term memory

**FIGURE 5:**
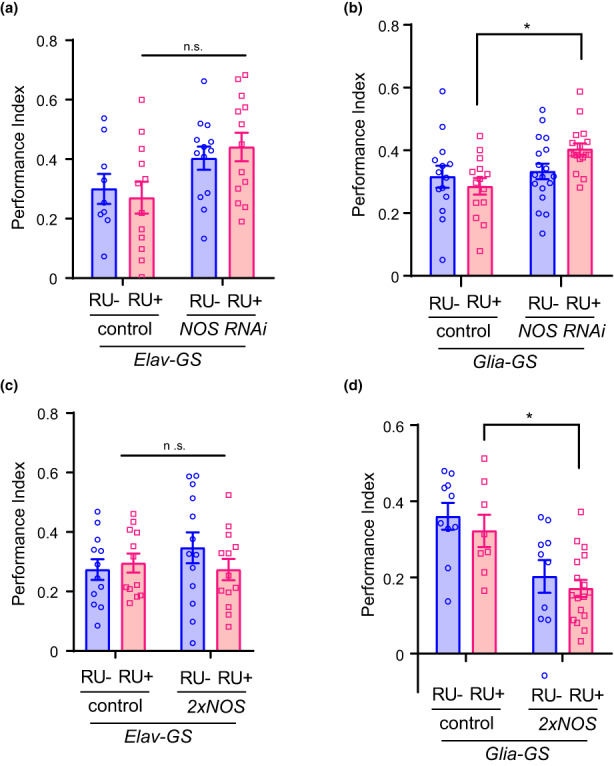
NOS expression in glia inhibits intermediate‐term memory. (a) Olfactory 3‐h memory assay with neuron‐specific knockdown of NOS using the GS system. No significant difference was observed in 3‐h memory between the control and experimental groups (two‐way ANOVA followed by post hoc Tukey's multiple comparisons test; *n* = 9, 12, 13, and 13 for control RU‐, control RU+, NOS^RNAi^ RU‐, and NOS^RNAi^ RU+ respectively. Two‐way ANOVA *F*_(1,43)_ = 7.974, *p* = 0.0072, post hoc Tukey's multiple comparisons test, p = 0.0602, control RU+ versus NOS^RNAi^ RU+). (b) Olfactory 3‐h memory assay with glia‐specific knockdown of NOS. Significant enhancement of 3‐h memory performance was observed in the experimental group (*Glia‐GS > NOS* ^*RNAi*^, RU+) compared with the control group (*Glia‐GS > GFP*, RU+) (two‐way ANOVA followed by post hoc Tukey's multiple comparisons test. *n* = 14, 14, 18, and 17 for control RU‐, control RU+, NOS^RNAi^ RU‐, and NOS^RNAi^ RU+ respectively. Two‐way ANOVA *F*_(1,59)_ = 6.882, *p* = 0.0111, post hoc Tukey's multiple comparisons test, *p* = 0.0109, control RU+ versus NOS^RNAi^ RU+). (c) Transient overexpression of NOS in neurons (*Elav‐GS > 2x NOS*) did not have a significant effect on ITM memory performance compared with the control group (*Elav‐GS > GFP*) (two‐way ANOVA followed by post hoc Tukey's multiple comparisons test; *n* = 12, 12, 13, and 13 for control RU‐, ontrol RU+, NOS RU‐, and NOS RU+ respectively. Two‐way ANOVA *F*_(1,46)_ = 0.4183, *p* = 0.5210). (d) Transient overexpression of NOS in glia (*Glia‐GS > 2x NOS*) significantly inhibited ITM compared with the control group (*Glia‐GS > GFP*) (two‐way ANOVA followed by post hoc Tukey's multiple comparisons test; *n* = 10, 8, 10, and 17 for control RU‐, control RU+, NOS RU‐, and NOS RU+ respectively. Two‐way ANOVA *F*_(1,41)_ = 19.77, *p* < 0.0001, post hoc Tukey's multiple comparisons test, *p* = 0.0158, control RU+ versus NOS RU+). n.s., not significant. Data are mean ± SEM for all

NOS has been reported to be broadly expressed in the adult brain (Kuntz et al., 2017). We examined whether the negative effect of NOS on ITM formation was induced by its expression in neurons or glia. First, NOS was knocked‐down in neurons only after the flies were fed RU using the GS system (*Elav‐GS*). When 5‐day‐old flies were fed RU for 5 days, memory performance at 3 h after conditioning was tested. A nonsignificant but upward trend in 3‐h memory performance was observed in the group of *NOS* ^*RNAi*^ expressing flies fed RU+ food compared with the control group of flies fed RU+ food (Figure 5a). Next, NOS was knocked down in glia only after the flies were fed RU using the GS system (*Glia‐GS*). A significant enhancement of 3‐h memory performance was observed in the group of flies fed RU+ food compared with the control group of flies fed RU‐ food, suggesting that NOS expression in glia had a negative effect on ITM formation (Figure 5b). Furthermore, NOS was overexpressed in neurons or glia to examine the effect of NOS on ITM. Although significant leaky expression was observed without RU feeding, *NOS* was overexpressed by 20 times or more by RU feeding (Figure S5). We found that overexpression of NOS in glia significantly inhibited ITM, while no significant difference in ITM was observed when NOS was overexpressed in neurons (Figure 5c,d). These data suggest that NOS expression mainly in glia inhibits ITM.

### 2.7 Inhibition of sGC and NOS enhances intermediate‐term memory in aged flies

**FIGURE 6:**
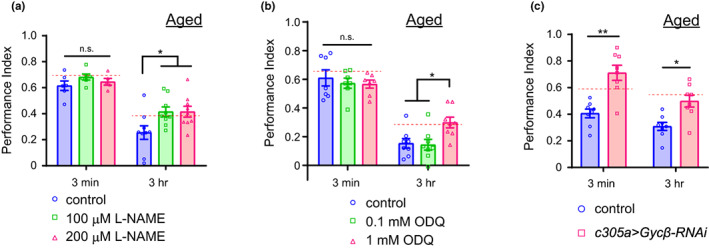
Inhibition of NOS or sGC enhances intermediate‐term memory in aged flies. (a) Olfactory memory assay in 30‐day‐old flies fed L‐NAME. L‐NAME administration at 100 or 200 μM concentration significantly improved 3‐h memory but did not affect 3‐min memory (two‐way ANOVA followed by post hoc Tukey's multiple comparisons test; 3 min, *n* = 6, 6, and 5; 3 h: *n* = 9 each for control, 100 μM L‐NAME, and 200 μM L‐NAME respectively. Twoway ANOVA *F*_(2,38)_ = 4.122, *p* = 0.0240. Post hoc Tukey's multiple comparisons test, *p = 0.0129, control versus 100 μM L‐NAME, *p* = 0.0127, control versus 200 μM L‐NAME). Red dot lines indicate the memory performance index of 3‐min or 3‐h respectively in a control group of young flies (Figure 4a). (b) Olfactory memory assay in 30‐day‐old flies fed ODQ. ODQ administration at 1 mM concentration significantly improved 3‐h memory but did not affect 3‐min memory (3 min, *n* = 7 for each data, *p* = 0.9708, Kruskal–Wallis test; 3 h, *n* = 8 for each data. *P* = 0.0155, Kruskal–Wallis test, post hoc Dunn's multiple comparisons test, *p = 0.0484, control versus 1 mM ODQ, *p = 0.0294, 0.1 mM ODQ versus 1 mM ODQ). Red dot lines indicate the memory performance index of 3‐min or 3‐h respectively in a control group of young flies (Figure 2a). (c) MB α′β′ neuron‐specific knockdown of *gycβ* significantly enhanced 3‐min and 3‐h memory in 30‐day‐old flies (two‐way ANOVA followed by post hoc Tukey's multiple comparisons test. *n* = 8 for each data. Two‐way ANOVA *F*_(1,28)_ = 33.95, *p* < 0.0001. Post hoc Tukey's multiple comparisons test, **p = 0.0001, 3 min control versus 3 min Gycβ^RNAi^, *p = 0.0183, 3 h control versus 3 h Gycβ^RNAi^). Red dot lines indicate the memory performance index of 3‐min or 3‐h respectively in a control group of young flies (Figure 3C). n.s., not significant. Data are mean ± SEM for all

Since aging increases the expression of *gyc β*, we examined whether inhibition of NO‐sGC signaling rescued age‐related memory impairment. Aged flies at 30‐days‐of‐age, which normally show ITM memory impairment but normal STM, were fed the NOS inhibitor L‐NAME for 18 h and their memory performance at 3 min and 3 h after conditioning was measured. A significant enhancement in memory performance at 3 h, but not at 3 min, after conditioning was observed in flies fed 100 or 200 μM L‐NAME (Figure 6a). The ITM enhancement induced by L‐NAME administration was observed at lower concentrations (100 μM) in 30‐day‐old flies (Figure 6a) than in 10‐day‐old flies (Figure 4a), suggesting that inhibition of NO‐sGC signaling enhanced ITM more sensitively in aged flies than in young flies. To examine when higher sensitivity to L‐NAME was observed earlier than at 30‐days‐of‐age, we examined the effect of L‐NAME on 3‐min and 3‐h memory in 20‐day‐old flies. The ITM was enhanced in 20‐day‐old flies when they were fed L‐NAME at 100 μM, suggesting that 20‐day‐old flies also had higher sensitivity to L‐NAME (Figure S6a). The enhancement of ITM was also observed when aged flies were fed 1 mM ODQ an sGC inhibitor (Figure 6b).

Furthermore, aged flies expressing *gyc β* ^*RNAi*^ in MB α′β′ neurons exhibited enhanced 3‐min and 3‐h memory performance (Figure 6c). To examine whether aging increases the expression of *gyc β*, we quantified GFP signals in young and aged flies expressing mCD8::GFP driven by *Gyc β ‐Gal4*. The GFP signals of MB α neurons and α′ neurons were increased in aged flies compared to that in young flies, indicating an age‐dependent increase in the expression of *gyc β* (Figure S6b). These results suggest that downregulated NOS or sGC function rescued memory performance in aged flies.

## 3 DISCUSSION

### 3.1 Candidate genes regulating age‐dependent memory impairment

Previous behavioral genetic screens of mutant or RNAi‐expressing flies to determine the regulation of olfactory memory have identified numerous pathways, including cAMP phosphodiesterase, calcium/calmodulin‐activated adenylyl cyclase, and the catalytic subunit of protein kinase A (PKA). Among these pathways regulating memory, several genes associated with synaptic transmission and neuronal structures have altered expression with aging and cause AMI (Jiang et al., 2001). However, the genes that regulate AMI remain largely unknown. Our comprehensive analysis of RNA‐seq data and genome‐wide RNAi screening data allowed us to identify candidate genes regulating AMI. We found that the *gycβ* gene, a subunit of sGC, negatively regulates ITM, raising the possibility that age‐related enhancement of the NO‐sGC pathway causes memory impairment. The list of other candidate genes whose expression was upregulated or downregulated with aging and potentially caused memory impairment (Tables S1 and S2) could provide a template for further genetic and pharmacological analyses of AMI.

### 3.2 Nitric oxide‐sGC pathway in memory formation

We found that both NOS and sGC, which are typically activated by NO to stimulate cGMP synthesis, were negative regulators of memory performance. Our findings are consistent with the proposed role of NO‐sGC in negatively regulating memory retention, that is, active forgetting, and in updating memory rapidly (Aso et al., 2019; Diaz et al., 2011; Wang et al., 2015), but contrast with its role in facilitating memory formation (Kemenes et al., 2002; Kuntz et al., 2017; Matsumoto et al., 2016; Müller, 1996). The NO‐sGC pathway is also known to form long‐term memory by activating the cAMP response element‐binding protein (CREB) in various animals (Harooni et al., 2009; Kemenes et al., 2002; Lu et al., 1999). These controversial results may be explained as follows: the NO‐sGC pathway may have a distinct effect on memory formation depending on the type of memory, area in the brain, and/or activity level of the NO‐sGC pathway.

A subunit of sGC, Gycβ, is strongly expressed in MB α′β′ neurons, which is required for memory acquisition and consolidation to form STM and ITM, and is weakly expressed in other types of MB neurons (Figure 3a, Figure S3a,b). Consistently, we found that knockdown of *gyc β* in MB α′β′ neurons enhanced STM and ITM (Figures 3c and 6c, Figure S4b,e). On the other hand, transient knockdown of *gycβ* in neurons using a gene‐switch system (Figure 1d) or the transient feeding of the sGC inhibitor ODQ (Figure 2a) enhanced ITM but did not change STM. While long‐term inhibition of NO/sGC signaling during the developmental stage using the *Gal4/UAS* system may lead to nonspecific enhancement of memory, transient inhibition of NO‐sGC signaling specifically enhances ITM. NO signaling plays critical roles in various developmental processes of the nervous system, including neurite patterning of the visual system and axon pruning/regrowth of mushroom body neurons (Cáceres et al., 2011; Johnston et al., 2011; Rabinovich et al., 2016). In particular, the inhibition of NO‐sGC signaling during development causes disorganization of neuronal projections and axon proceeding, especially in the *Drosophila* visual system (Gibbs et al., 2001; Gibbs & Truman, 1998). However, it is still unclear whether the long‐term inhibition of NO‐sGC signaling from the developmental stage is the actual cause of disorganization of neuronal projections, leading to nonspecific enhancement of memory. Furthermore, as aging specifically affects ITM (Tamura et al., 2003; Tonoki & Davis, 2012), it leaves open whether enhanced expression of *gycβ* in MB α′β′ neurons is directly responsible for AMI.

NOS has been reported to be broadly expressed in the adult brain (Kuntz et al., 2017). We found that NOS expression in glia impaired ITM, while NOS knockdown in glia enhanced ITM (Figure 5), suggesting that NO synthesis in glia‐regulated ITM. A recent study has shown that NO produced in glia regulates circadian locomotor behavior (Kozlov et al., 2020). Thus, it is possible that memory processes are modulated by NO in glia through changes in circadian behavior. However, other possibilities for the critical site of NO synthesis to regulate memory have been proposed. The localization of NO in ellipsoid body ring neurons activates the CREB pathway to form visual working memory (Kuntz et al., 2017). NO acts as a cotransmitter in a subset of dopaminergic neurons to negatively regulate memory retention (Aso et al., 2019). Future studies will reveal the effect of NO on memory processes in neurons other than the glial cells.

### 3.3 sGC regulates age‐related memory impairment

As previously shown, aging impairs ITM formation. We found that age‐related impairment of ITM is reversed by pharmacological inhibition of NOS and sGC and by knockdown of *gyc β* in MB α′β′ neurons (Figure 6). RNA‐seq data showed that the expression of *gyc β* increased with aging. These lines of evidence suggest that the overproduction of NO or activation of NO‐sGC signaling can cause age‐related memory impairment. A previous study in crickets showed that age‐related memory impairment in LTM was rescued by NO donor or cGMP analog injection (Matsumoto et al., 2016), which contrasts with our conclusion showing the reverse of memory impairment by the inhibition of NOS or sGC. This may also explain that the NO‐sGC‐cGMP pathway has a distinct effect on memory formation in STM/ITM and LTM. The synthesis of NO by NOS plays a significant role in the pathological processes. Although it is unclear whether aging induces overproduction of NO, excessive synthesis of NO has been suggested to cause DNA damage, protein modifications, and cell toxicity, leading to neuronal cell death (Wang et al., 2015) and neurodegenerative diseases, including Parkinson's disease (Kanao et al., 2012).

Biochemical studies have shown that PKA can enhance the NO‐induced activity of sGC by phosphorylating sGC (Zhang et al., 2002). Behavioral analysis has shown that an agedependent increase in PKA activity in MBs causes memory impairment via glial dysfunction (Yamazaki et al., 2007, 2014). These studies and our observations raise the possibility that aging could impair ITM not only by increasing the expression of a subunit of sGC but also by phosphorylating sGC by increasing PKA activity.

### 3.4 Experimental procedures

#### 3.4.1 Fly stocks and genetics

Fly crosses were raised at 25°C and 70% relative humidity under a 12‐h light–dark cycle. The wild‐type strain was *Canton‐S*. Approximately 250 flies that were born in 2–3 days were raised in food bottles and transferred to fresh bottles every 3 or 4 days until the age required for each experiment. 10‐ or 30‐day‐old flies were used as young or aged flies, respectively. For the GS experiment, RU486 was administered at a final concentration of 200 μM. *UAS‐GFP*, *UAS‐Gycβ‐RNAi* ^*HMJ22589*^, *UAS‐Cp190‐RNAi* ^*HMJ02105*^, *UAS‐amon‐RNAi* ^*GL01217*^, *UAS‐SA‐RNAi* ^*HMS00272*^, *UAS‐NOS‐RNAi* ^*GLC01867*^, *UAS‐2xdNos, UAS‐mCD8::GFP, c305a‐Gal4*, *MB005B‐Gal4, MB463B‐Gal4, Gyc β ‐Gal4* ^*MI01568*^, *Gycbeta100B* ^*MI08892‐GFSTF.2*^, and *Elav‐GS* were obtained from the Bloomington *Drosophila* Stock Center of Indiana University. *Glia‐GS (GSG3285‐1)* was a gift from Dr. Haig Keshishian (Yale University).

#### 3.4.2 Learning and memory assay

The learning and memory assays were performed under dim red light at 23°C and 70% relative humidity. Standard aversive olfactory conditioning experiments were performed as previously described (Tonoki & Davis, 2012). Approximately 50 flies were placed in an electric shock tube, where they were exposed to odors and electric shocks. The flies were exposed to 1 min of an odor paired with 12 pulses of electric shock at 90 V (CS+), which was followed by 30 s of air and then 1 min of a second odor without shock (CS−). For the conditioning odors, we bubbled fresh air through 3‐octanol at a concentration of 0.12% and benzaldehyde at concentrations of 0.07% or 0.08% in mineral oil. To measure early memory, we immediately transferred the flies into a T‐maze, where they were given 2 min to choose between an arm with the CS+ odor and an arm with the CS− odor. To test memory performance 3, 6, or 9 h after conditioning, the flies were placed back into food vials at 23°C until the memory performance test. For the behavioral measurements, two groups of flies were trained simultaneously with two different odors used as CS+. The one‐half performance index (PI) for each odor was calculated as (the number of flies that chose CS− minus the number that chose CS+)/(the number of flies that chose CS− plus the number of flies that chose CS+). The overall PI was then calculated as the average of the two one‐half PIs for each odor. This method balanced out naïve odor biases.

#### 3.4.3 Drug treatments

ODQ (1H‐[1,2,4]oxadiazolo[4,3‐a]quinoxalin‐1‐one, Tokyo Chemical Industry) was dissolved in dimethyl sulfoxide (DMSO) and mixed with a 5% sucrose solution to a final concentration of 0.1 mM or 1 mM. For L‐NAME (Nω‐nitro‐L‐arginine methyl ester, Sigma‐Aldrich), a 100 μM or 200 μM solution was prepared in a 5% sucrose solution. Filter paper cut to a size of 8 × 4.3 cm^2^ was soaked in 1.5 ml of solution in each vial. Flies were raised in these vials for 18 h before training.

#### 3.4.4 Olfactory acuity

The odor avoidance tests were performed for 2 min in a T‐maze by allowing naïve flies at the indicated ages to choose between an odor in mineral oil on one side and fresh air in mineral oil without an odor on the other side. The avoidance index was calculated as follows: (the number of flies that chose the fresh‐air side minus the number that chose the odor side)/(the number of flies that chose the fresh‐air side plus the number that chose the odor side).

#### 3.4.5 Sensory acuity

The shock avoidance tests were performed for 2 min in a T‐maze by allowing naïve flies at the indicated ages to choose between one side with 12 pulses of electric shock at 90 V and the other side without an electric shock. An avoidance index was calculated as follows: (the number of flies that chose the “shock−” side minus the number that chose the “shock+” side)/(the number of flies that chose the “shock−” side plus the number that chose the “shock+” side).

#### 3.4.6 Immunohistochemistry

We dissected and fixed fly brains with 4% paraformaldehyde and incubated them at 4°C for 5 days with mouse anti‐Fasciclin II (FasII) (1D4, DSHB, 1:50), 9.4A anti‐Trio‐S (DSHB, 1:4), nc82 (DSHB, 1:20), and anti‐GFP (ab13970, 1:2000). Images were acquired with a Leica TCS SP8 confocal microscope. Image stacks were collapsed into a two‐dimensional maximum projection image with ImageJ (NIH). Regions of interest (ROIs) were drawn corresponding to the mushroom body compartments detected by a nc82 antibody and the GFP fluorescence or the relative GFP fluorescence in each region was measured using ImageJ.

#### 3.4.7 RNA‐sequencing

*Canton‐S* male flies were collected at 10‐ or 30‐days‐of‐age, and the heads of 100 male flies were used for RNA extraction. Triplicate biological samples were collected. RNA was isolated from each sample using RNAiso Plus (Takara‐bio). cDNA libraries were generated using the Agilent Strand Specific RNA prep kit and run on a HiSeq 2500 sequencing system (Illumina). Analysis of differentially expressed genes was performed using the R package DESeq2. The data set was deposited in the DNA Data Bank of Japan, DDBJ (ID: SSUB004439).

#### 3.4.8 qPCR

Total RNA was extracted from 50 heads of the indicated flies with Sepasol RNA I Super G (Nacalai), and reverse‐transcription reactions were performed with ReverTra Ace (Toyobo). qPCR was performed with Power SYBR Green PCR Master Mix (Thermo Fisher Scientific) and a LightCycler 96 system (Roche Life Science). The expression levels were normalized against those of *rp49*. Melting temperature analysis confirmed that each primer pair produced a single PCR product. The following primers were used. *gyc β*: 5′‐gacggggacaaagagcagaa‐3′ and 5′‐atcaggtggaaggggaacac‐3′. *Nos*: 5′‐gaacccacgtgtggaagaag‐3′ and 5′‐cgatgtaaatttcacagcccta‐3′. *rp49*: 5′‐atcggttacggatcgaacaa‐3′ and 5′‐gacaatctccttgcgcttct‐3′.

#### 3.4.9 Experimental design and statistical analysis

All statistical tests and data analyses were performed using the Prism7 software (Graphpad). The statistical tests used and the value of n are indicated in figure legends. The comparisons were considered to be statistically significant when *p* < 0.05. Collected data were tested whether they were sampled from a Gaussian distribution by D'Agostino & Pearson or Shapiro–Wilk normality test. If the data were considered to be sampled from a Gaussian distribution, they were compared by two‐tailed unpaired t test or one‐way ANOVA followed by Tukey's multiple comparisons test. Otherwise, they would be compared by two‐tailed Mann–Whitney test or Kruskal–Wallis test followed by Dunn's multiple comparisons test. Memory scores were displayed as mean ± SEM. Other data including qPCR data were displayed as mean ± SD.

## AUTHOR CONTRIBUTIONS

Saki Nagai, Zhihua Yu, Tong Yue, Sizhe Lyu, Xue Hou, Kotomi Onuki, Kaho Yabana, and Ayako Tonoki. performed experiments, analyzed data, and prepared figures. Hiroki Takahashi analyzed data and prepared figures. Ayako Tonoki, Saki Nagai, Zhihua Yu, and Motoyuki Itoh planned the study and wrote the paper. All authors edited the paper and approved the final manuscript.

## CONFLICT OF INTEREST

The authors declare that there is no conflict of interest.

## Supporting information


Appendix S1

## Data Availability

The data that support the findings of this study are available from the corresponding author upon reasonable request.
