# Supplementary material for: Nitric oxide‐soluble guanylyl cyclase pathway as a contributor to age‐related memory impairment in Drosophila
Source: Aging Cell. 2022 Aug 13;21(9):e13691. doi: 10.1111/acel.13691 (PMC9470885; doi:10.1111/acel.13691)
Supplement: Supplementary file 1 — Appendix S1 [file ACEL-21-e13691-s001.pdf]

Supplementary file

Nitric oxide-soluble guanylyl cyclase pathway as a contributor to age-related memory impairment in *Drosophila*

Ayako Tonoki[1], Saki Nagai[1], Zhihua Yu[1], Tong Yue[1], Sizhe Lyu[1], Xue Hou[1], Kotomi Onuki[1], Kaho Yabana[1], Hiroki Takahashi[2], Motoyuki Itoh[1]

1. Department of Biochemistry, Graduate School of Pharmaceutical Sciences, Chiba University, Chiba 260-8675, Japan

2. Medical Mycology Research Center, Chiba University

Corresponding Author: Ayako Tonoki

Department of Biochemistry, Graduate School of Pharmaceutical Sciences, Chiba University, Chiba 260-8675, Japan

Tel: +81-43-226-2890

Fax: +81-43-226-2890

E-mail: tonoki@chiba-u.jp

Supplementary information

Figure S1. Neuron-specific knockdown of *gycβ* enhances intermediate-term memory (Related to Figure 1).

(A, B) Venn diagrams showing the overlap between two different gene lists of RNA-sequencing data and genome-wide RNAi screen data: four genes are negative memory regulators with age-dependent upregulation of gene expression (A) and fifty-four genes are positive memory regulators with age-dependent downregulation of gene expression (B). (C) Each candidate gene was transiently knocked-down in neurons only after the flies were fed RU486 (RU) using *Elav-GS*. When 5-day-old flies were fed RU-containing food for 5 days, memory performance was tested at 3 hr after conditioning. Enhancements in 3-hr memory were observed in flies expressing $cp190^{RNAi}$ or $gyc\beta^{RNAi}$ in neurons compared with control. (n = 18, 18, 18, 18, 10, 11, 10, 10, 12, and 12 for control RU-, control RU+, $cp190^{RNAi}$ RU-, $cp190^{RNAi}$ RU+, $Gyc\beta^{RNAi}$ RU-, $Gyc\beta^{RNAi}$ RU+, $amon^{RNAi}$ RU-, $amon^{RNAi}$ RU+, SA^{RNAi} RU-, and SA^{RNAi} RU+ respectively. Two-way ANOVA $F_{(4, 127)} = 8.882$, $p < 0.0001$ for row factor and $F_{(4, 127)} = 10.13$, $p = 0.0018$ for column factor. post hoc Tukey's multiple comparisons test, *$p < 0.05$, $cp190^{RNAi}$ RU- versus $cp190^{RNAi}$ RU+; **$p < 0.05$, control RU-/RU+ versus $Gyc\beta^{RNAi}$ RU+.)

Data are mean ± SEM for (C).

Figure S2. Neuron-specific knockdown of *gycβ* (Related to Figure 1).

(A) qPCR shows that the silencing efficiency of the knockdown of *gycβ* genes in neurons was approximately 50 % in the flies expressing $gyc\beta^{RNAi\text{-}HMJ22589}$ ($gyc\beta^{RNAi}$). (n = 3, 3, 3, and 2 for control RU-, control RU+, $Gyc\beta^{RNAi}$ RU-, and $Gyc\beta^{RNAi}$ RU+ respectively. Two-way ANOVA $F_{(1, 7)} = 16.57$, $p = 0.0047$, post hoc Tukey's multiple comparisons test, *$p = 0.0320$, control RU+ versus RNAi RU+). (B) Shock avoidance test in *gycβ* knockdown flies. There was no significant difference between the experimental group and the control group in the avoidance of electric shock (n=10 for each data. $t_{(18)} = 0.2334$, $p = 0.8181$). (C) Odor avoidance test in *gycβ* knockdown flies. There was also no significant difference between the experimental group and the control group in the avoidance of odors, Oct and Benz (n=10 for each data. Two-way ANOVA $F_{(1, 36)} = 2.55$, $p = 0.1191$). (D) 3-hr memory after cold shock treatment given 2 hr after conditioning. Transient and neuron-specific knockdown of *gycβ* (*Elav-GS*> $gyc\beta^{RNAi}$) significantly enhanced 3-hr memory after cold shock compared to the control group (Two-way ANOVA followed by *post hoc* Tukey's multiple comparisons test. n = 15, 14, 10, and 12 for control RU-, control RU+, RNAi RU-, and RNAi RU+ data respectively. Two-way ANOVA $F_{(1, 47)} = 10.14$, $p = 0.0026$, post hoc Tukey's multiple comparisons test, *$p < 0.05$,

control RU+ versus RNAi RU+.).

n.s.: not significant. Data are mean ± SEM for (B-D) and mean ± SD for (A).

Figure S3. Expression of *gycβ* in MB α′β′ neurons (Related to Figure 3).

(A) Fly brains expressing membrane-tethered GFP (*UAS-mCD8::GFP*) driven by *Gycβ-Gal4*^{MI01568} (green) were colabeled with an anti-FasII antibody (magenta) to mark MB αβ neurons. Weak expression of *gycβ* in MB αβ neurons was observed. (B) Expression of *Gycβ* labeled with Gycβ-EGFP in flies carrying the Gycβ[MI08892-GFSTF.2] construct. Gycβ-EGFP signals are shown in the anterior and posterior confocal sections. Gycβ-EGFP signals in MB α′ and β′ neuron in control flies (left panels) were markedly reduced in flies with expression of *gycβ*^{RNAi} in MB α′β′ neurons (right panels). (C) Gycbeta-EGFP signals in the α′ and β′ were significantly reduced in flies expressing *gycβ*^{RNAi} in MB α′β′ neurons using *c305a-Gal4* compared with that in the control group. (α lobe, n = 11 and 13 for control and *gycβ*^{RNAi}, $t_{(22)} = 0.2234$, $p = 0.8253$; β lobe, n = 9 and 13 for control and *gycβ*^{RNAi}, $t_{(20)} = 1.995$, $p = 0.0599$; α′ lobe, n = 11 and 14 for control and *gycβ*^{RNAi}, $t_{(23)} = 2.439$, $*p = 0.0228$; β′ lobe, n = 11 and 14 for control and *gycβ*^{RNAi}, $t_{(23)} = 10.94$, $*p < 0.0001$, unpaired *t* test).

Data are mean ± SD.

Figure S4. Expression of *gycβ* in MB α′β′ neurons (Related to Figure 3).

(A) Gycbeta-EGFP signals in the α′ and β′ were significantly reduced in flies expressing *gycβ*^{RNAi} in MB α′β′ neurons using *MB005B-Gal4* compared with that in the control group. (α lobe, n = 4 and 3 for control and *gycβ*^{RNAi}, $t_{(5)} = 1.482$, $p = 0.1984$; β lobe, n = 4 and 3 for control and *gycβ*^{RNAi}, $t_{(5)} = 0.9719$, $p = 0.3757$; α′ lobe, n = 4 and 3 for control and *gycβ*^{RNAi}, $t_{(5)} = 2.572$, $*p = 0.0499$; β′ lobe, n = 4 and 3 for control and *gycβ*^{RNAi}, $t_{(5)} = 7.086$, $*p = 0.0009$, unpaired *t* test). (B) MB α′β′ neuron-specific knockdown of *gycβ* (*MB005B>Gyc*β^{RNAi}) significantly enhanced 3-hr memory compared with that in the control (*MB005B>GFP*) (3 min, n = 7 for each data, $t_{(12)} = 2.178$, $p = 0.0501$; 3 hr, n = 11 and 9 for control and *gycβ*^{RNAi}, $t_{(18)} = 2.536$, $*p = 0.0207$, unpaired *t* test). (C) Shock avoidance test in *gycβ* knockdown flies. There was no significant difference between the experimental group and the control group in the avoidance of electric shock (n = 7 for each data, U = 24, $p = 0.4418$, Mann-Whitney U test). (D) Odor avoidance test in *gycβ* knocked-down flies. There was no significant difference between the experimental group and the control group in the avoidance of odors, Oct and Benz. (Oct, n = 8 and 7 for control and *gycβ*^{RNAi}, $t_{(13)} = 0.2384$, $p = 0.8153$; Benz, n = 8 for each data, $t_{(14)} = 0.2496$, $p = 0.8065$). (E) MB α′β′ neuron-specific knockdown of *gycβ* (*MB463B>Gyc*β^{RNAi}) significantly enhanced 3-min and 3-hr memory compared with control (*MB463B>GFP*) (3 min, n = 9 and 7 for control and *gycβ*^{RNAi}, $t_{(14)} = 7.1$, $*p < 0.0001$; 3 hr, n

= 13 for each data, $t_{(24)}$ = 3.603, *p = 0.0014, unpaired *t* test). (F) Shock avoidance test in *gycβ* knockdown flies. There was no significant difference between the experimental group and the control group in the avoidance of electric shock (n = 6 for each, $t_{(10)}$ = 0.05725, p = 0.9555, unpaired *t* test). (G) Odor avoidance test in *gycβ* knocked-down flies. There was no significant difference between the experimental group and the control group in the avoidance of odors, Oct and Benz. (Oct: n = 8 for each, $t_{(14)}$ = 2.693, *p = 0.0175; Benz, n = 6 for each data, $t_{(10)}$ = 0.2811, p = 0.7844).

n.s.: not significant. Data are mean ± SEM for all.

Figure S5. Overexpression of NOS in glia (Related to Figure 5).

qPCR shows that *NOS* was overexpressed by 20 times or more by RU feeding in *Glia-GS>NOS* flies compared to control flies. (Two-way ANOVA followed by *post hoc* Tukey's multiple comparisons test. n = 3 for each data. Two-way ANOVA $F_{(1, 8)}$ = 375.4, p < 0.0001. post hoc Tukey's multiple comparisons test, *p < 0.0001).

Data are mean ± SD.

Figure S6. Inhibition of NOS enhances intermediate-term memory in aged flies (Related to Figure 6).

(A) Olfactory memory assay in 20-day-old flies fed L-NAME. L-NAME administration at 100 μM concentration significantly improved 3-hr memory but did not affect 3-min memory (Two-way ANOVA followed by *post hoc* Tukey's multiple comparisons test; 3min, n = 7 for each data; 3 hr, n = 11, 12, and 13 for control, 100 μM L-NAME, and 200 μM L-NAME, respectively. Two-way ANOVA $F_{(2, 51)} = 8.134$, $p = 0.0009$, post hoc Tukey's multiple comparisons test, *p=0.0406, 3 hr-control versus 3 hr-100 μM L-NAME). Data are mean ± SEM. (B) Quantification of GFP signal in 10-day-old (10d) and 30-day-old (30d) flies expressing mCD8::GFP driven by *Gycβ-Gal4*. The GFP signals of MB α neurons and α' neurons were increased in aged flies compared with young flies (Two-way ANOVA followed by *post hoc* Tukey's multiple comparisons test; n = 9 and 7 for 10d and 30d, respectively. Two-way ANOVA $F_{(1, 28)} = 23.62$, $p < 0.0001$, post hoc Tukey's multiple comparisons test, *$p = 0.0393$, MB α' 10 d versus 30 d; *$p = 0.0021$, MB α 10 d versus 30 d; **$p < 0.0001$, MB α' 10 d versus MB α 10 d). Data are mean ± SD.

Figure. S1

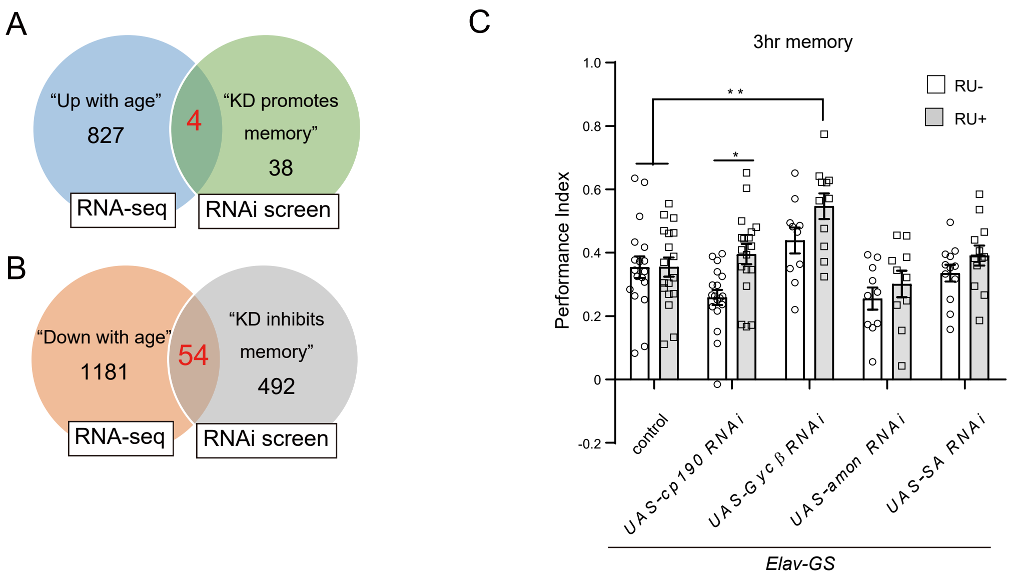
A
"Up with age"
827
4
"KD promotes memory"
38
RNA-seq
RNAi screen
B
"Down with age"
1181
54
"KD inhibits memory"
492
RNA-seq
RNAi screen
C
3hr memory
Performance Index
1.0
0.8
0.6
0.4
0.2
0
-0.2
RU-
RU+
**
*
control
UAS-cp190 RNAi
UAS-Gycβ RNAi
UAS-amon RNAi
UAS-SA RNAi
Elav-GS

Figure. S2

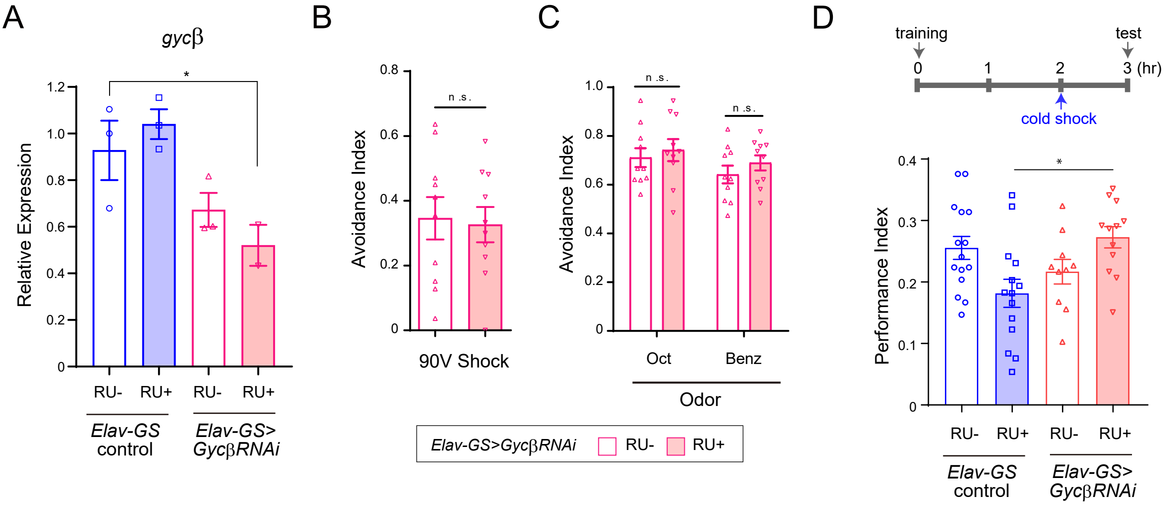

A
gycβ
Relative Expression
*
RU-
RU+
RU-
RU+
Elav-GS control
Elav-GS> GycβRNAi
B
Avoidance Index
n.s.
90V Shock
C
Avoidance Index
n.s.
n.s.
Oct
Benz
Odor
Elav-GS>GycβRNAi
RU-
RU+
D
training
test
0
1
2
3 (hr)
cold shock
Performance Index
*
RU-
RU+
RU-
RU+
Elav-GS control
Elav-GS> GycβRNAi

Figure. S3

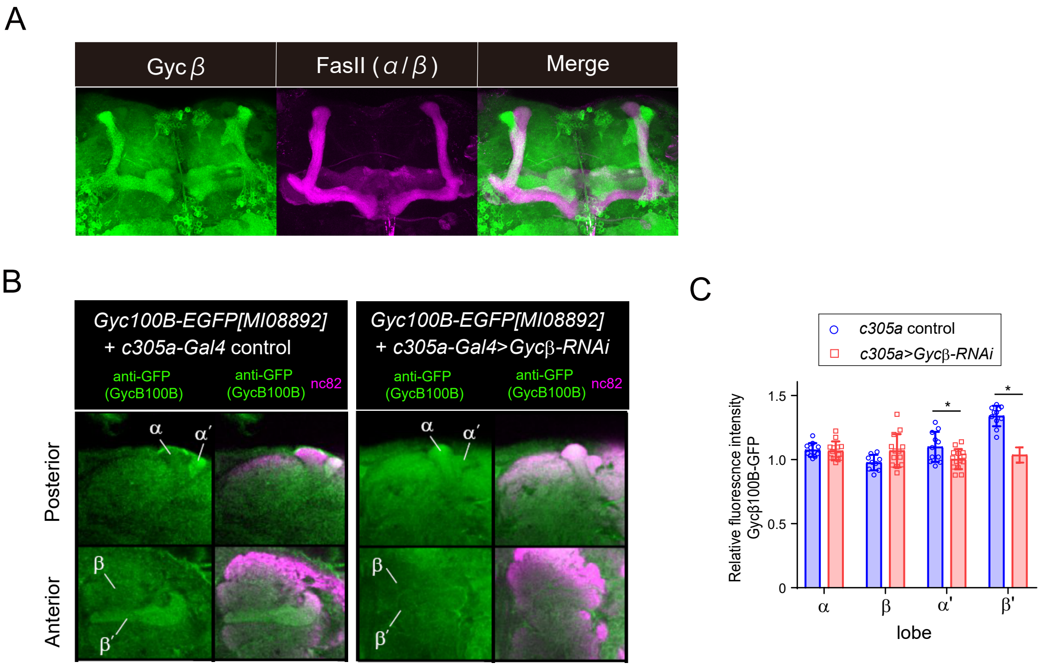

A
Gyc β
FasII (α / β)
Merge
B
Gyc100B-EGFP[MI08892]
+ c305a-Gal4 control
anti-GFP (GycB100B)
anti-GFP (GycB100B) nc82
Gyc100B-EGFP[MI08892]
+ c305a-Gal4>Gycβ-RNAi
anti-GFP (GycB100B)
anti-GFP (GycB100B) nc82
Posterior
Anterior
α
α′
β
β′
C
c305a control
c305a>Gycβ-RNAi
Relative fluorescnce intensity Gycβ100B-GFP
1.5
1.0
0.5
0
*
*
α
β
α'
β'
lobe

Figure. S4

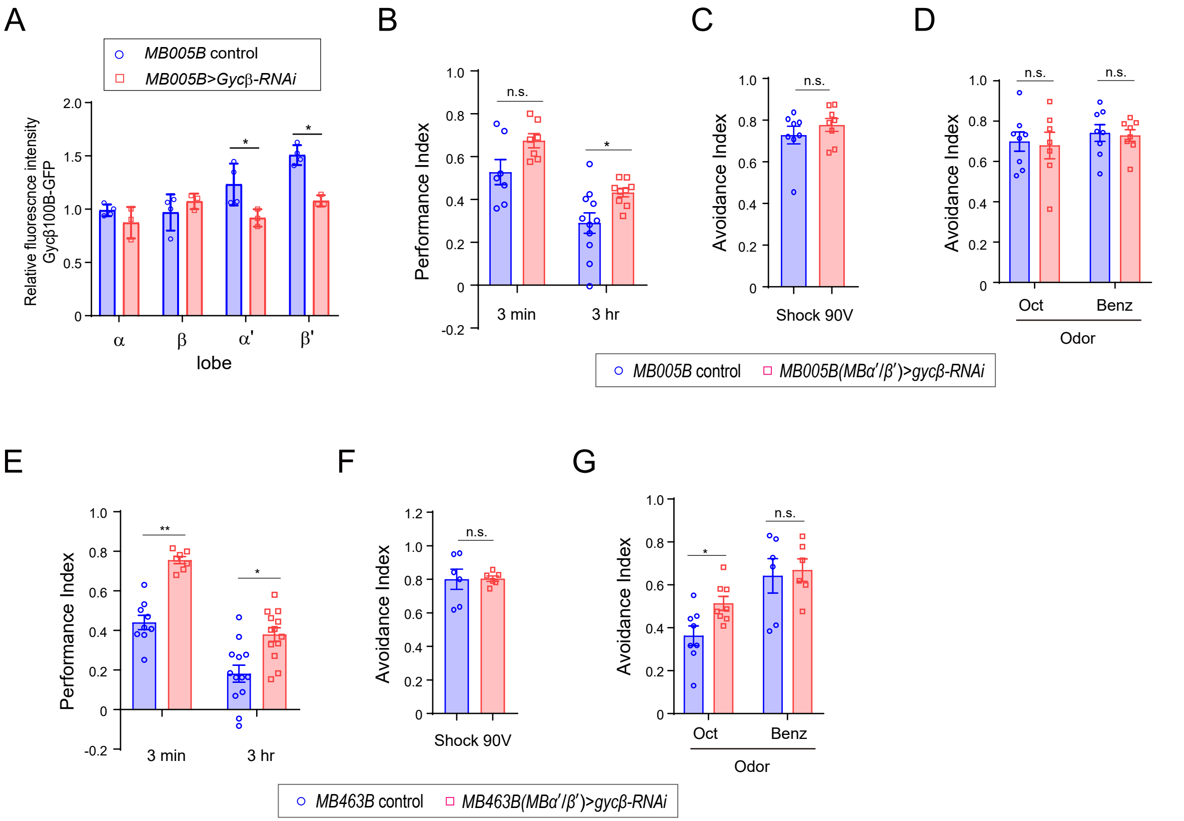
A
MB005B control
MB005B>Gycβ-RNAi
Relative fluorescnce intensity Gycβ100B-GFP
2.0
1.5
1.0
0.5
0
*
*
α
β
α'
β'
lobe
B
Performance Index
1.0
0.8
0.6
0.4
0.2
0
-0.2
n.s.
*
3 min
3 hr
C
Avoidance Index
n.s.
Shock 90V
D
Avoidance Index
n.s.
n.s.
Oct
Benz
Odor
MB005B control
MB005B(MBα′/β′)>gycβ-RNAi
E
Performance Index
**
*
3 min
3 hr
F
Avoidance Index
1.2
n.s.
Shock 90V
G
Avoidance Index
*
n.s.
Oct
Benz
Odor
MB463B control
MB463B(MBα′/β′)>gycβ-RNAi

Figure. S5

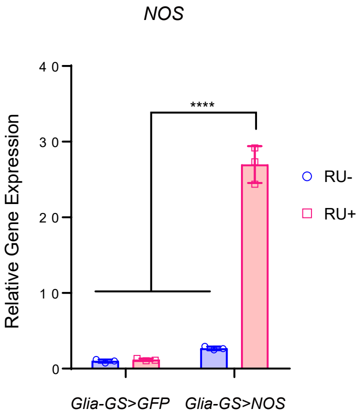
NOS
Relative Gene Expression
40
30
20
10
0
****
RU-
RU+
Glia-GS>GFP
Glia-GS>NOS

Figure. S6

A

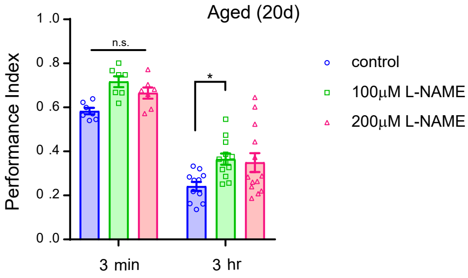
Aged (20d)
Performance Index
1 .0
0 .8
0 .6
0 .4
0 .2
0 .0
n.s.
*
3 min
3 hr
control
100μM L-NAME
200μM L-NAME

B

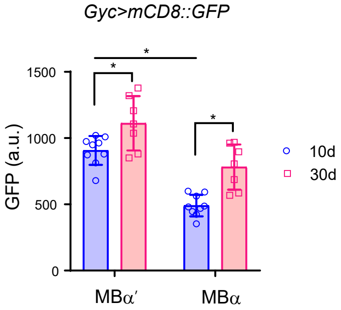
Gyc>mCD8::GFP
GFP (a.u.)
1500
1000
500
0
*
*
*
MBα′
MBα
10d
30d

Table S1. List of genes that are negative-memory regulators with age-dependent upregulating gene expression

Gene ID	CG number	Gene Name	BaseMean	log2FoldChange	pvalue
FBgn0000283	CG6384	Cp190	4086.674094	0.345788762	4.86E-06
FBgn0013973	CG1470	Gycbeta100B	5417.554561	0.248444822	2.10E-07
FBgn0020616	CG3423	SA	1523.413465	0.220247667	0.001137266
FBgn0023179	CG6438	amon	13543.6522	0.277229669	5.25E-08

Table S2. List of genes that are positive-memory regulators with age-dependent downregulating gene expression

Gene ID	CG number	Gene Name	BaseMean	log2FoldChange	pvalue
FBgn0000422	CG10697	Ddc	10209.29924	-0.161249	0.009236328
FBgn0003423	CG1417	sIgA	13788.04981	-0.212593	4.45E-06
FBgn0003475	CG10076	spir	7847.563597	-0.18385	1.68E-04
FBgn0004623	CG8770	Gbeta76C	17878.20036	-0.370805	2.09E-11
FBgn0004903	CG6354	Rb97D	2092.141754	-0.292246	9.59E-06
FBgn0005561	CG11049	sv	972.4737607	-0.72329	8.20E-10
FBgn0011758	CG5529	B-H1	210.5815042	-0.445442	6.31E-04
FBgn0014396	CG3234	tim	37545.90429	-0.349188	6.18E-11
FBgn0016075	CG16858	vkg	1642.218135	-0.363091	1.67E-07
FBgn0021979	CG3082	l(2)k09913	6423.14735	-0.273099	6.30E-06
FBgn0024963	CG7535	GluClalpha	16440.20957	-0.164068	0.004641559
FBgn0025549	CG1659	unc-119	2733.079954	-0.186491	0.00488067
FBgn0027885	CG6582	Aac11	8888.388485	-0.238343	5.22E-08
FBgn0028699	CG7499	Rh50	698.5451648	-0.977746	1.83E-14
FBgn0029801	CG15771	CG15771	3226.250201	-0.221155	2.47E-05
FBgn0029819	CG3016	Usp30	1284.807535	-0.211158	0.005081925
FBgn0029830	CG14447	Grip	249.8349895	-0.363879	0.00510487
FBgn0030087	CG7766	CG7766	6517.94836	-0.273341	1.01E-07
FBgn0030668	CG8128	CG8128	500.6648223	-0.341756	4.91E-04
FBgn0030670	CG9245	Pis	9257.448139	-0.315721	6.65E-07
FBgn0030895	CG7135	CG7135	2082.340104	-0.351498	1.21E-04
FBgn0031971	CG7224	Sirup	1536.481931	-0.475816	9.13E-09
FBgn0031998	CG8451	SLC5A11	700.5792629	-0.618638	1.47E-08
FBgn0032021	CG7781	CG7781	7826.394016	-0.231729	2.72E-06
FBgn0032729	CG10639	L2HGDH	547.6830392	-0.339418	9.66E-04
FBgn0033434	CG1902	CG1902	1446.078606	-0.187497	0.004702246
FBgn0034051	CG8295	Mlf	6958.798379	-0.237696	5.05E-04
FBgn0034576	CG9350	ND-B14.7	2431.818654	-0.29144	0.004488762
FBgn0034902	CG5532	CG5532	285.6281657	-0.388102	0.003379101
FBgn0035526	CG1316	CG1316	4604.477238	-0.216033	1.05E-05
FBgn0035695	CG10226	CG10226	2060.916751	-0.373209	9.76E-06
FBgn0036043	CG8177	Ae2	13322.09774	-0.446129	2.84E-15
FBgn0036428	CG9238	Gbs-70E	4244.302115	-0.732464	3.84E-11
FBgn0037138	CG7145	P5CDh1	13591.10786	-0.174893	2.94E-04
FBgn0037607	CG8036	CG8036	3863.597691	-0.467017	3.35E-05

FBgn0037655	CG11984	Kcmf1	5879.36327	-0.138537	0.005413205
FBgn0038610	CG7675	CG7675	960.7764541	-0.444555	2.18E-06
FBgn0039132	CG5864	AP-1sigma	2364.048263	-0.176463	0.002752215
FBgn0039635	CG11876	Pdhb	3143.669328	-0.617765	1.41E-14
FBgn0039748	CG15529	CG15529	333.3041799	-0.519183	4.12E-06
FBgn0051005	CG31005	qless	772.5825998	-0.28746	0.001408794
FBgn0051352	CG31352	Unc-115a	2230.638195	-0.177975	0.0042323
FBgn0052000	CG32000	anne	22296.54772	-0.199862	0.006889574
FBgn0052672	CG32672	Atg8a	11275.86514	-0.249744	4.32E-06
FBgn0085405	CG34376	CG34376	1344.174565	-0.477632	7.53E-12
FBgn0086673	CG13272	CG13272	42.24566195	-1.278612	3.91E-10
FBgn0259111	CG42253	Ndae1	4515.954776	-0.16187	0.003318863
FBgn0260743	CG18347	GC1	1836.300869	-0.244418	5.70E-05
FBgn0261477	CG5186	slim	2604.24922	-0.19727	6.26E-04
FBgn0261955	CG3861	kdn	12888.21548	-0.572736	4.44E-31
FBgn0262476	CG43066	CG43066	9067.295112	-0.383012	1.19E-12
FBgn0263199	CG5288	Galk	1208.850134	-0.647784	4.61E-07
FBgn0263776	CG43693	CG43693	1318.304619	-0.471205	2.65E-08
FBgn0264308	CG43778	CG43778	8208.064806	-0.314511	5.36E-05
